# Cardiac Resynchronization Therapy in Cardiogenic Shock: A Case-Based Discussion

**DOI:** 10.7759/cureus.18157

**Published:** 2021-09-21

**Authors:** Francisco J Somoza-Cano, Juan F Toledo, Ramses Amaya-Handal, Abdul Rahman Al Armashi, Francisco R Somoza

**Affiliations:** 1 Internal Medicine, St. Vincent Charity Medical Center, Cleveland, USA; 2 Internal Medicine, Hospital CEMESA, San Pedro Sula, HND; 3 Interventional Cardiology, Hospital CEMESA, San Pedro Sula, HND; 4 Interventional Cardiology, Cardio Center, San Pedro Sula, HND

**Keywords:** multiorgan failure, cardiac electrophysiology, cardiology, interventional cardiology, cardiac resynchronization therapy (crt), cardiogenic shock, critical care, internal medicine

## Abstract

Cardiac resynchronization therapy (CRT) has consistently proven its capability to improve the left ventricular ejection fraction (LVEF). The benefits and indications for this therapy have been elucidated in current heart failure guidelines. However, it remains a topic of discussion if there is a role for it in acute heart failure syndromes (AHFSs). We present the case of a 55-year-old male with a medical history of alcohol-induced cardiomyopathy presenting with a new left bundle branch block, a widened QRS (154 ms), and cardiogenic shock (CS). After a lack of improvement with optimal medical management, CRT was used as a last resort. After implantation, the patient had a satisfactory clinical course and the LVEF improved. At the four-month follow-up, he underwent an outpatient transthoracic echocardiogram with further augmentation of his LVEF, improvement of his functional class, and no reported acute heart failure events. This case illustrates a potential therapeutic option for CS with a widened QRS. Prospective trials should include AHFSs to clarify the utility of CRT in this patient population.

## Introduction

Cardiac resynchronization therapy (CRT) has been shown to reduce the morbimortality in chronic, stable heart failure patients with reduced left ventricular ejection fraction (LVEF) and a widened QRS [1]. However, the major trials have excluded patients with acute heart failure syndromes (AHFSs) [2]. Therefore, the potential risks and benefits of these devices in AHFSs are largely unknown. Furthermore, cardiogenic shock (CS) is a common cause of mortality in patients presenting with myocardial infarction (MI). The management remains challenging despite advances in therapeutic options proven by the unchanged six- to twelve‐month mortality over the past two decades [3]. Further therapeutic options are needed, and CRT might be an appropriate adjuvant alternative.

## Case presentation

**Figure 1:**
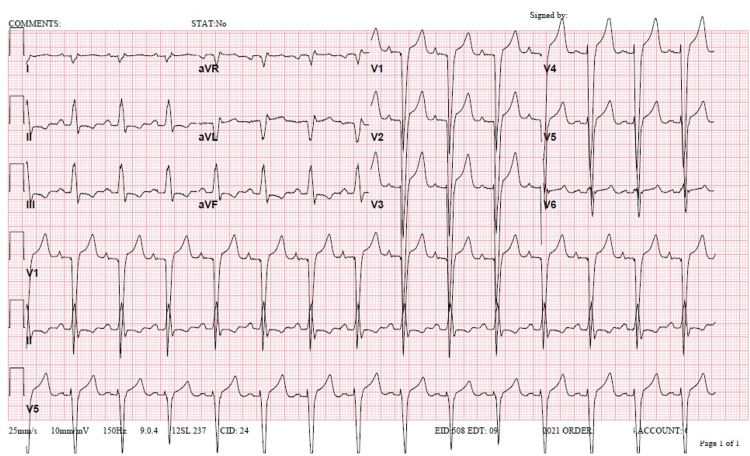
Electrocardiogram on admission. A 12-lead electrocardiogram was obtained on admission. A sinus rhythm with fusion complexes can be observed. There is a right axis deviation with a left bundle branch block. QRS was 154 ms, heart rate was 82 beats per minute, PR interval was 180 ms, and QTc was 500 ms.

**Figure 2:**
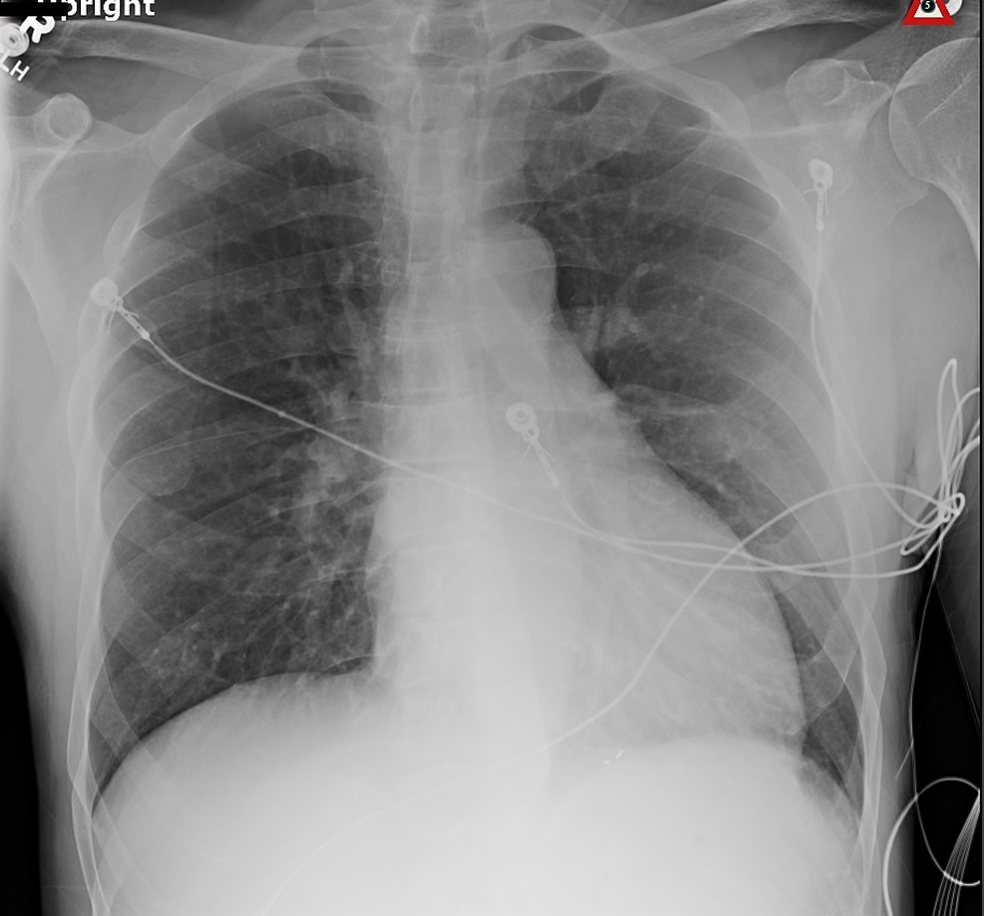
Chest X-ray findings. A single anteroposterior portable chest X-ray was obtained. No acute intrathoracic processes were observed.

A 55-year-old male with a medical history of alcohol-induced cardiomyopathy and alcoholic cirrhosis presented to the emergency department complaining of typical chest pain of 30-minute duration. He reported that the last alcoholic drink was consumed over two years ago. The New York Heart Association (NYHA) functional class was IV before the acute presentation. Physical examination revealed a blood pressure of 89/57 mmHg, heart rate of 106 beats per minute, respiratory rate of 23 breaths per minute, pulse oximetry of 92% at room air, and a temperature of 36.6°C. Initial workup was remarkable for troponin I of 0.068 ng/mL (reference range: 0.01-0.045 ng/mL), creatinine of 1.3 mg/dL (reference range: 0.3-1.5 mg/dL) with a baseline of 1 mg/dL, and blood urea nitrogen (BUN) of 29 mg/dL (reference range: 5-24 mg/dL). Aspartate aminotransferase was 150 U/L (reference range: 15-37 U/L), alanine aminotransferase was 73 U/L (reference range: 13-61 U/L), and lactic acid was 3.4 mmol/L (reference range: 0.8-2.0 mmol/L). Arterial blood gases showed primary metabolic acidosis compensated with respiratory alkalosis. Complete blood count, electrolytes, and coagulation times were unremarkable (Table 1). An electrocardiogram (EKG) revealed a new left bundle branch block (LBBB) with a QRS of 154 ms (Figure 1). In addition, a chest X-ray showed no acute processes (Figure 2).

**Table 1:** Laboratory findings.

Lab finding	Result	Unit
Complete blood count		
White blood cells	8.94 × 10^3^	K/uL
Red blood cells	4 × 10^6^	M/uL
Hemoglobin	13.5	g/dL
Hematocrit	41	%
Mean corpuscular volume	93	fL
Mean corpuscular hemoglobin	31.3	pg
Mean corpuscular hemoglobin concentration	35.9	g/dL
Platelets	204 × 10^3^	K/uL
Complete metabolic panel		
Sodium	141	mmol/L
Potassium	3.88	mmol/L
Chloride	110	mmol/L
Calcium	8.51	mg/dL
Magnesium	1.9	mg/dL
Creatinine	1.3	mg/dL
Blood urea nitrogen	29.9	mg/dL
Aspartate aminotransferase	150	U/L
Alanine aminotransferase	73	U/L
Albumin	4	g/dL
Prothrombin time	16	seconds
Partial thromboplastin time	29.6	seconds
International normalized ratio	1.2	IU
Troponin I	0.068	ng/mL
Lactic acid	3.4	mmol/L
Arterial blood gas		
pH	7.36	
pCO_2_	38	mmHg
pO_2_	94	mmHg
HCO_3_	22	mmol/L
Base excess	-4	meq/L
Temperature	37	Celsius
SO_2_	97	%
FiO_2_	30	%

**Figure 3:**
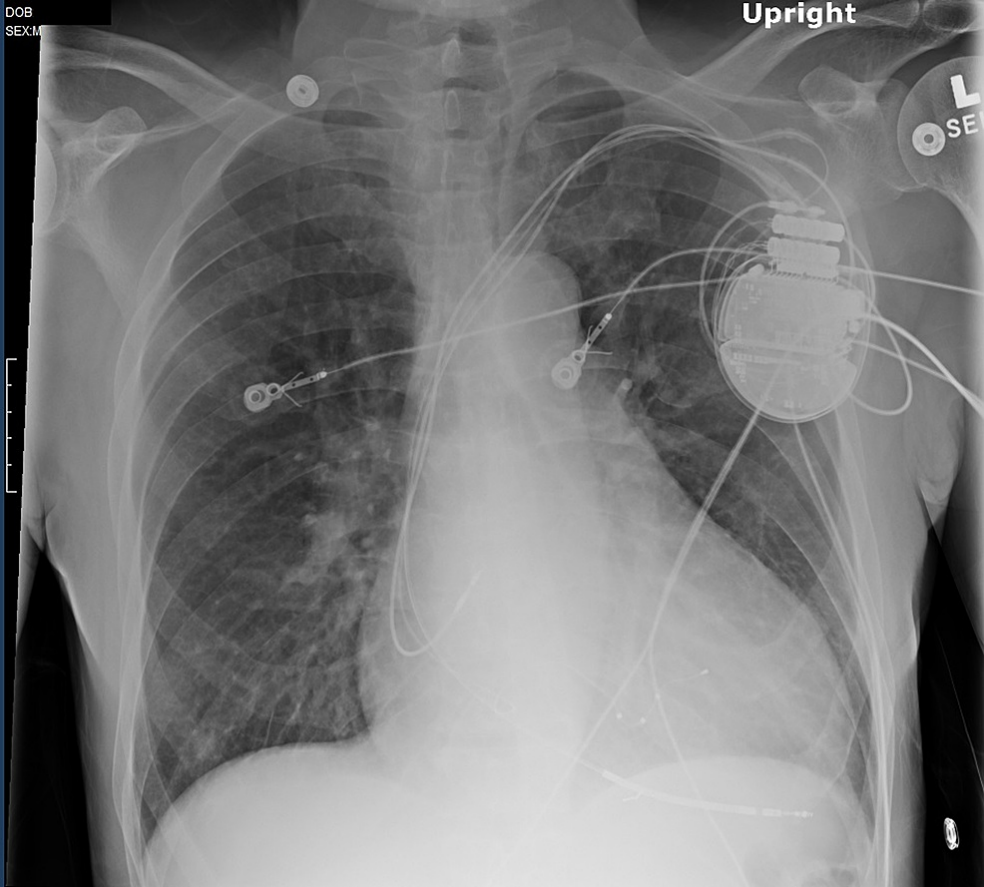
Post-procedure chest X-ray. A portable anteroposterior chest x-ray was obtained after the procedure. An implantable cardioverter-defibrillator pace/sense/defibrillator lead was placed in the right ventricle septal apex where appropriate function was demonstrated. The other leads can be seen at the right atrium and the coronary venous sinus. The leads were sutured to the pectoralis muscle with a suture sleeve. Adequate hemostasis was ensured and the pocket was flushed with an antibiotic solution. The leads were connected to the generator and the system was placed into the pre-pectoral pocket. The wound was closed with layers of absorbable suture. The procedure was tolerated well and there were no complications.

He was medically treated as an ST-segment elevation myocardial infarction (STEMI) and taken for an emergent percutaneous coronary intervention. A 90% stenosis of the left anterior descending artery was noted for which a 2.5 mm × 28 mm drug-eluting stent was implanted. The pulmonary capillary wedge pressure was 27 mmHg (reference range: 4-12 mmHg) and LVEF (by modified Simpson method) was 10%. Dobutamine and norepinephrine were started at the catheterization laboratory and the patient was transferred to the Intensive Care Unit. After a poor response, levosimendan was added to the medication regimen. However, 36 hours later, the patient continued with worsening signs of hypoperfusion given by delayed capillary refill time (over five seconds), worsening lactic acid (5.7 mmol/L), worsening transaminitis, and acute kidney injury stage 3 (creatinine of 4.0 mg/dL). The patient required mechanical ventilation and continued with increasing pressure support demands. The Sequential Organ Failure Assessment (SOFA) score was 11 points. Mechanical circulatory support devices were not available. Therefore, the decision was made to implant a CRT defibrillator device (Figure 3).

**Figure 4:**
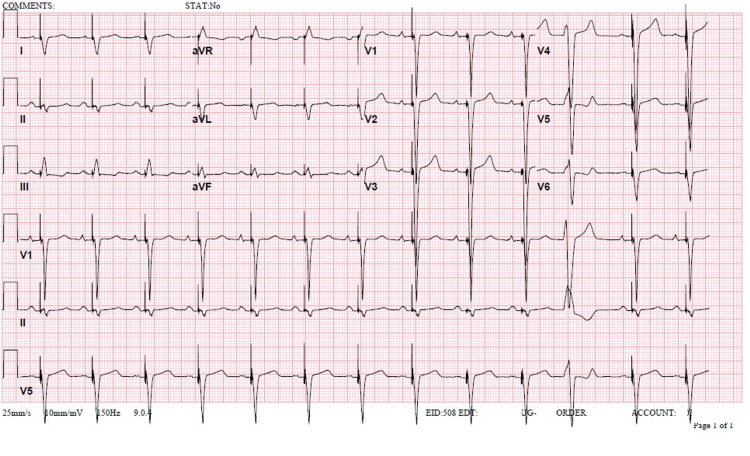
Electrocardiogram on follow-up. An electrocardiogram was obtained during outpatient follow-up. A QRS of 130 ms was seen with a ventricular-paced rhythm, Furthermore, the patient had a heart of 77 beats per minute, a PR of 176 ms, and a QTc of 513 ms.

Afterward, the patient remained in a critical condition but his clinical status started to improve over time. A new transthoracic echocardiogram (TTE) showed an LVEF of 15-20%. The patient was discharged on optimal medical management for heart failure with reduced ejection fraction (HFrEF), which included metoprolol succinate, spironolactone, sacubitril/valsartan, and close outpatient follow-up. Sodium-glucose co-transporter-2 inhibitors were withheld due to borderline blood pressure. A TTE obtained four months after the initial presentation showed an ejection fraction of 20-25%. NYHA functional class was III-IV and the patient denied acute heart failure events. An outpatient EKG showed a ventricular-paced rhythm (Figure 4).

## Discussion

Dyssynchronous ventricular contraction with delayed electrical activation (e.g., widened QRS) undoubtedly impairs the LVEF. This is due to the hemodynamic necessity for uniform cardiac contraction to maintain adequate perfusion pressure over time. The off-timing of atrial and ventricular contraction, mismatch contraction between the left and right ventricle, and the impaired coordinated contraction of the left ventricle itself skew the normal heart physiology, prompting a mechanism for resynchronization [4].

CRT is a proven mechanism that reduces morbimortality and induces left ventricular reverse remodeling in patients with compromised LVEF (<35%), widened QRS, and stable, chronic heart failure. However, even though there is a consistency among international guideline recommendations, there are certain patient populations where the eligibility is highly debatable [1,5]. Even though our patient had worsening heart failure, a widened QRS, and a reduced LVEF, randomized clinical trials (RCTs) for CRT have failed to include patients with AHFSs. The high burden of complications and short life expectancy have made the trials disregard such patients [2].

Moreover, chronic alcohol consumption remains a leading cause of secondary cardiomyopathy. The prevalence of alcohol-induced cardiomyopathy in patients with heart failure varies from 4% to 40% with a similar prevalence among men and women. However, the disease burden is much higher in men, with a male-to-female ratio for hospital admission of 9:1. The main age group affected is 45-59 years. Moreover, there is evidence of a correlation between HFrEF and cirrhosis. In one patient cohort, cirrhosis occurred in 43% of patients with cardiomyopathy compared to 6% in patients without cardiomyopathy, as seen in our patient. The outcomes of acute heart failure events are significantly worsened when there is associated cirrhosis, even if compensated [6,7].

Furthermore, in patients presenting with MI, CS remains a significant cause of mortality. CS is a complex syndrome caused by altered hemodynamics and characterized by a low cardiac output that very frequently leads to organ failure and death with a mortality rate of around 40% [8]. Acute MI leads to the majority of patients who present with CS\, as seen in our case. The American Heart Association guidelines for the treatment of CS after a STEMI specify the risk factors that increase the probability of CS. Age more than 70 years, systolic blood pressure less than 120 mmHg, sinus tachycardia or sinus bradycardia, and increased time of onset are the most common risk factors associated with this condition [9]. Our patient had two of the aforementioned risk factors. Moreover, once CS ensues, the standard of care remains inotropic support [8]. Our patient received intravenous dobutamine followed by levosimendan after dobutamine proved insufficient. Dobutamine improves cardiac function by its inotropic and inodilator properties [8]. Levosimendan is a calcium sensitizer with inotropic and vasodilatory actions mediated by the sensitization of contractile proteins to calcium, opening of potassium channels, and inhibition of phosphodiesterase-3 [10]. Although not approved by the Food and Drug Administration, the European Heart Association guidelines have adopted this medication due to its positive clinical outcomes in recent RCTs [5,10].

Furthermore, if the patient persists with CS, the Society for Cardiovascular Angiography and Interventions/American College of Cardiology/Heart Failure Society of America guidelines recommend MCS devices (i.e., intra-aortic balloon pump, extracorporeal membrane oxygenation devices) [11-13]. Many hospitals have adopted a multidisciplinary shock team approach with in-hospital guidelines for these devices in the acute management of CS [11]. However, even with these adjuvants therapies, the long-term mortality for patients with CS remains unchanged [3]. Moreover, the medical costs associated with this approach are out of reach for community medical centers similar to the one our patient presented to. Therefore, exploring long-term therapeutic options from the initial presentation needs to be considered.

The lack of therapeutic options in a patient with worsening acute heart failure, a widened QRS, and multiorgan failure with a SOFA score of 11 (50% mortality) made CRT an appropriate adjuvant alternative to be considered [14]. Moreover, Piccini et al. reported that, even though major CRT trials have not studied CRT in AHFSs, a significant proportion of these devices are implanted in the acute in-patient setting. The rationale behind this decision needs to be explored with prospective trials but likely reflects the necessity for additional options in the setting of a life-threatening medical condition [15]. Rescue CRT remains debatable, but different heart teams worldwide have used this option as a last resort [16,17].

## Conclusions

This case illustrates a possible adjuvant alternative in the management of cardiogenic shock. The prolongation of the patient’s life span, stabilization of his LVEF, and the improvement in his NYHA functional class make this type of therapy an option to be considered. Quality prospective research must be conducted to elucidate the potential benefits of this therapy in AHFSs.
